# Large energy storage density performance of epitaxial BCT/BZT heterostructures via interface engineering

**DOI:** 10.1038/s41598-019-53358-0

**Published:** 2019-11-14

**Authors:** Amrit P. Sharma, Dhiren K. Pradhan, Sangram K. Pradhan, Messaoud Bahoura

**Affiliations:** 1 Center for Materials Research, Norfolk State University, 700 Park Avenue, Norfolk, VA 23504 USA; 2 Geophysical Laboratory, Carnegie Institution for Science, Washington D.C., 20015 USA

**Keywords:** Energy science and technology, Nanoscience and technology

## Abstract

We grew lead-free BaZr_0.2_Ti_0.8_O_3_ (BZT)/Ba_0.7_Ca_0.3_TiO_3_ (BCT) epitaxial heterostructures and studied their structural, dielectric, ferroelectric and energy density characteristics. The BZT/BCT epitaxial heterostructures were grown on SrRuO_3_ (SRO) buffered SrTiO_3_ (STO) single crystal substrate by optimized pulsed laser deposition (PLD) technique. These high-quality nanostructures exhibit high dielectric permittivity (∼1300), slim electric field-dependent polarization (P-E) curve with high saturation polarization (∼100 µC/cm^2^) and low remnant polarization (∼20 µC/cm^2^) through interface engineering to develop new lead-free ferroelectric system for energy storage devices. We observe an ultrahigh discharge and charge energy densities of 42.10 and 97.13 J/cm^3^, respectively, with high efficiency, which might be highly promising for both high power and energy storage electrical devices.

## Introduction

Energy crisis will be the world’s number one problem due to the ever-growing energy demand and escalating energy prices. The demand of energy is expected to double worldwide in next thirty years^1^, resulting in an accelerated depletion of natural resources. Fossil fuels, such as coal, which have negative environmental impacts including global warming, greenhouse effect, acid rain, and air pollution^2,3^, are expected to be fully depleted in next thirty years^1^. Therefore, the current research is focused on alternate cleaner and more sustainable energy resources, such as solar, wind, water, heat, vibration, stress, magnetic field, etc^4^. The energy produced from these resources is mainly electrical in nature, which is often challenging to store. By solving the problem of energy storage with highly efficient devices, an avenue to transition from dependence on non-renewable energy to an era of renewable energy will be formed.

In the recent years, dielectric capacitors with high-energy storage densities are the optimal option among currently available energy storage devices, such as batteries, dielectric capacitors, super-capacitors, and fuel cells^5–7^. In dielectric capacitors, the dielectric layer stores electrostatic energy in the form of electric displacement induced by an applied electric field. In other words, dielectric capacitors have a unique energy storage mechanism with an ultrafast charging/discharging process and high power density capabilities^8^, which are critical for modern electrical and electronic power systems including hybrid electric vehicles, electrical weapons systems and high-frequency inverters^9–12^. However, the energy density of dielectric capacitors (~2 J/cm^3^) is relatively low as compared to fuel cells or Li-ion batteries (>20 J/cm^3^)^13,14^. Therefore, it is imperative to improve the energy density of dielectric materials. In fact, all dielectrics capacitors including thin films, bulk ceramics and polymers show high power density because of their extremely fast charge/discharge speed compared to all other existing energy storage devices^15,16^. In addition, due to massive structural defects like pores, voids and impurities, the bulk ceramics possess very low energy density and breakdown strengths^17,18^. However, thin-film capacitors only exhibit better performance in energy density because they, simultaneously, possess high polarization as well as high breakdown strengths due to the high quality and epitaxial growth of dielectric thin film. The continuing drive towards miniaturization of electronic circuits and devices is a motivating factor for the design and development of suitable dielectric thin-films as energy storage materials^19^.

The energy density (W) and the efficiency (η) of the dielectric capacitors are determined by polarization (P) versus applied electric field (E) curve via

1 $$W={\int }_{Pr}^{Pmax}\,E\,dP$$

2 $${\rm{\eta }}=\frac{W}{W+Wloss}\times 100 \% $$

where P_max_, P_r_ and W_loss_ are the maximum polarization, remnant polarization and the energy storage loss, respectively^20–22^. From Eq. (1), high P_max_, low P_r_ and large breakdown field are required to achieve high energy density^23,24^. Relaxor-ferroelectric and antiferroelectric materials are very promising materials for obtaining high energy density because of their high P_max_ and low P_r_^10^. However, antiferroelectric materials possess high energy dissipation, and hence low η^25^. Therefore, relaxor-ferroelectrics with slim P-E hysteresis loops are mostly preferred for high energy density capacitor applications^3,26^.

Recently, very high energy density of up to 62 J/cm^3^ at an applied field of 3.13 MV/cm has been reported in lead-based relaxor-ferroelectric thin films^27^. Nevertheless, the lead-based materials are unfriendly for human health and environment and thus their applications will be seriously limited in the future. Many lead-free materials have been systematically investigated, and some promising results have been reported to date. Lead-free Hf_0.3_Zr_0.7_O_3_ thin films exhibit a large recoverable energy density of ~40 J/cm^3^ with efficiency ~50%^28^. Most lead-free materials have modified BaTiO_3_-based relaxor-ferroelectric thin films. Ultra-high efficiency of ~ 81% with recoverable energy density of ~37 J/cm^3^ has been reported in 0.88BaTiO_3_-0.12Bi (MgTiO_3_) films^29^. Some lead-free materials contain Na, Bi and K, however these materials have very poor densification because they evaporate at high temperature^30^. Similarly, efficiency of 72.3% with recoverable energy density of 52.4 J/cm^3^ has been reported recently in Ba_0.7_Ca_0.3_TiO_3_-BaZr_0.2_Ti_0.8_TiO_3_ supper-lattices^31^. In the existing literature, high-energy storage performance of lead-free relaxor-ferroelectric thin films are obtained with a large applied field, but most of the devices cannot withstand such a large electric field for practical applications. It is imperative to look for materials that have high-energy storage density at a moderate electric field and are competitive with lead-based materials. Several approaches have been reported to solve these problems, such as compositions near phase boundaries^32^, via domain engineering^26^, utilizing space charges^33^ or interface effect^34,35^ and the addition of a dead layer^36^.

In this paper, we present an interface engineering approach on a new lead-free relaxor system, BCT/BZT heterostructure to observe slim P-E loop with high P_max_, low P_r_, and high breakdown field strength, which has been acknowledged a promising alternative to lead-based materials. It is known that the isovalent substitution of Ba^2+^ by Ca^2+^ and Ti^4+^ by Zr^4+^ (i.e. BCT and BZT, respectively) can transform micro size ferroelectric domains into highly dynamic polar nano-regions (PNRs), exhibiting ferroelectric to relaxor-ferroelectric transition^31,37,38^. Due to their relaxor characteristics, BCT and BZT are very promising for industrial applications; numerous efforts have been carried out, recently, to understand the physics of relaxor behavior on these materials. It is now commonly accepted that the PNRs are responsible for unusual behavior of relaxor-ferroelectrics^39,40^. In relaxor-ferroelectric materials at low temperature, some atoms might occupy off-center displacement caused by a local random electric field due to charge being unbalanced inside a unit cell and forming short-range polar clusters which improve the effective dipole moment significantly^40^.

**Figure 1:**
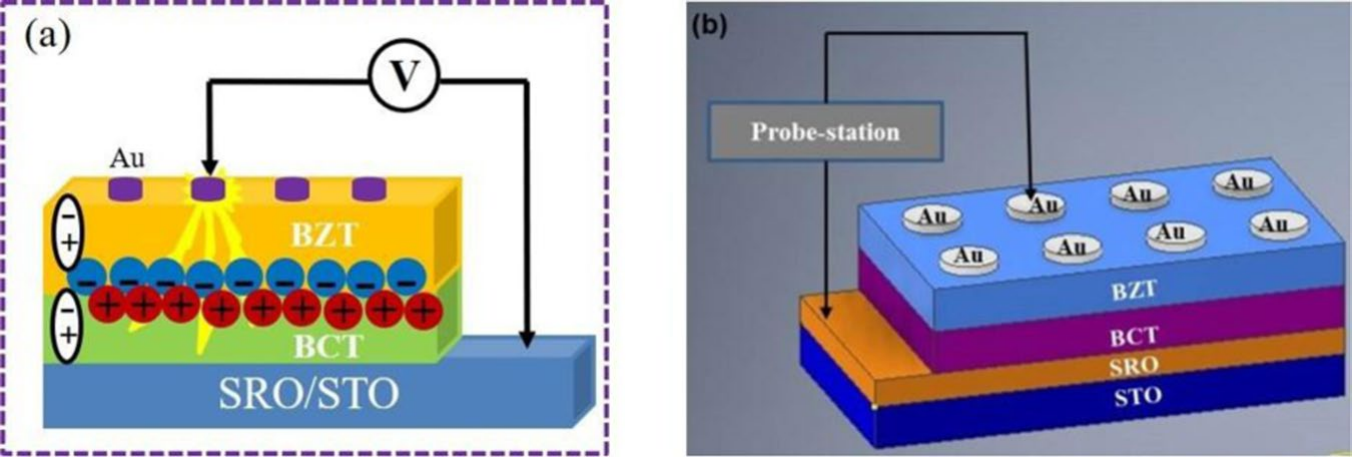
Schematic diagram of interface engineering (**a**) and fabrication of BZT/BCT heterostructures (**b**).

As an artificial material, ferroelectric multilayers/superlattices exhibit excellent performances, as compared to their single layer counter part because of their unique oxygen octahedral shape and the interfaces between the adjacent layers^34,41^. The interface acts as a medium, which generates space charges (schematically shown in Fig. 1a ^35^); and these accumulated interfacial charges could serve as effective charge traps for injected electrons from the metal electrodes under the application of a high electric field^35,42^. All these results demonstrate that through epitaxial growth and careful interface engineering, the BCT/BZT multilayer system enhances its electrical insulation, improves the polarization and hence energy density performances.

## Experimental Details

Indigenously prepared targets were used for the fabrication of the epitaxial thin films of BZT, BCT, and their multilayers heterostructures on SRO buffered STO (100) single crystal substrate using pulsed laser deposition (PLD) with a KrF excimer laser^34^. A 50 nm thick SRO, as a bottom electrode, was deposited at a substrate temperature of 800 °C in an oxygen partial pressure of 20 mTorr with a laser energy density of ~2 J/cm^2^. It was annealed in the same oxygen atmosphere of 300 Torr for 30 minutes at the same temperature and then cooled down to ambient temperature. The thin films of BZT, BCT, and their multilayers structures were grown at the same substrate temperature of 800 °C under an oxygen partial pressure of 100 mTorr with the same laser energy. Later, the single layers and multilayers thin films were annealed under the same circumstances as the SRO and cooled down to room temperature slowly. For comparison of all physical properties, the thickness of all the thin films BZT, BCT and BZT/BCT were kept fixed at ~260 nm.

The phase and crystallographic orientations of BZT, BCT and BZT/BCT thin films were analyzed via x-ray diffraction (XRD) using a commercial Rigaku (D/Max-2200TB) diffractometer, operated at 40 mA and 40 kV using CuK_α_ radiation at a scan rate of 0.125° per min over the wide angular range (2θ) of 20° to 80° at room temperature. The thicknesses of all thin films were measured using a profilometer (DektakXT, Bruker Corporation). For electrical characterization, circular gold (Au) top electrodes of radius ~100 μm with thickness ~60 nm were prepared by thermal evaporation technique utilizing a metal shadow mask. The electrical and dielectric properties were measured using a semiconductor characterization system (Keithley 4200) and an impedance analyzer (HP4294A), respectively. Ferroelectric hysteresis (P-E) loops were measured at room temperature using a Precision Materials Analyzer (Radiant Technologies Inc.). All the graphing and data analysis were performed using OriginPro.

## Results and Discussion

### Structural studies

**Figure 2:**
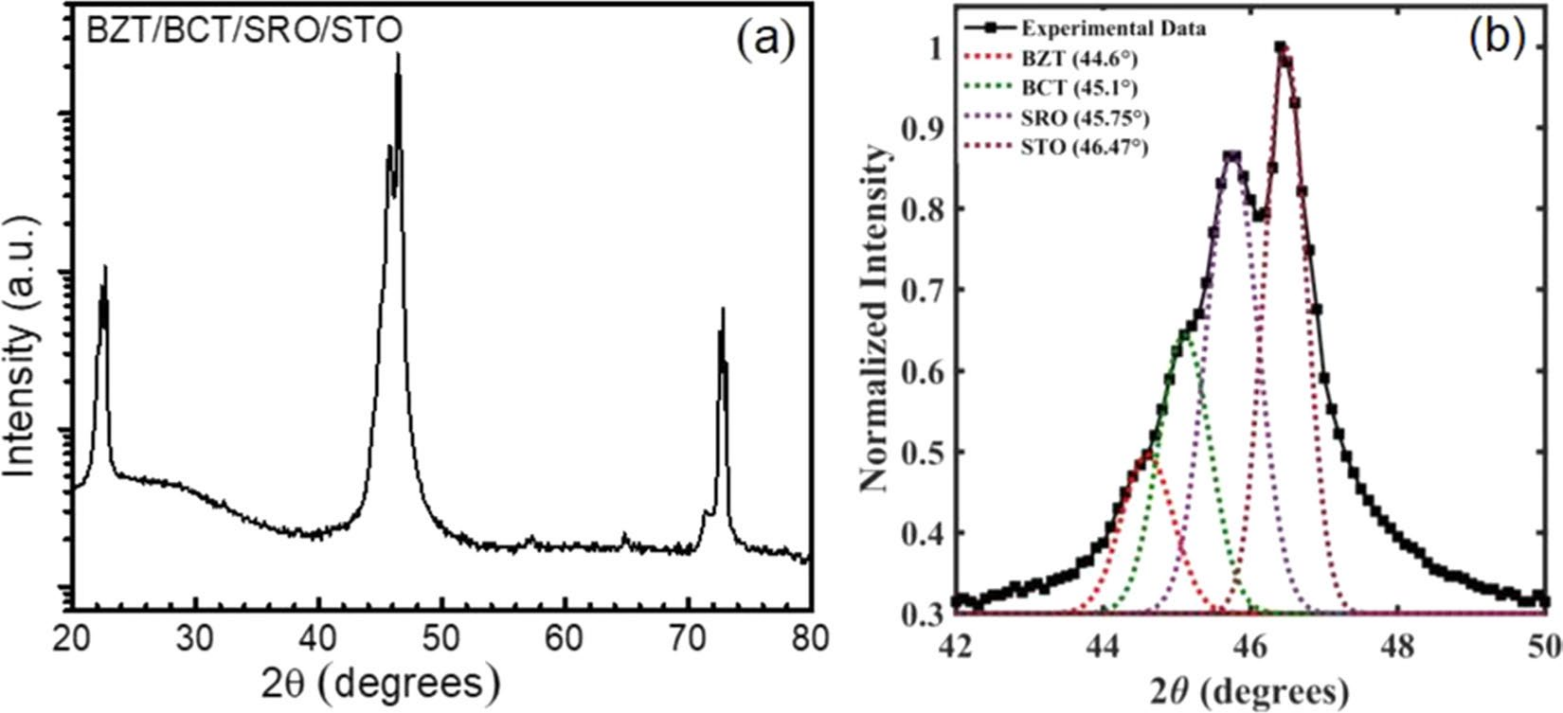
Room temperature XRD patterns of BZT/BCT heterostructure on SRO buffered STO (100) substrate (**a**), Zoom in on (200) peaks of BZT, BCT, SRO and STO (**b**).

The schematic diagram of BZT/BCT heterostructure grown on SRO buffered STO (100) single crystal substrate is as shown in Fig. 1(b). To check the epitaxial nature of the BZT/BCT films on SRO buffered STO (100) substrate, high-resolution XRD measurement of the heterostructure was performed and shown in figure below. Figure 2(a) shows the XRD patterns of BZT/BCT bilayer heterostructure recorded at large angle x-ray scans from 20° to 80° at room temperature. The diffraction peaks from the substrate, SRO, and the pseudo cubic reflections (*l00*) from materials (BZT/BCT) confirm that these heterostructures are highly oriented and epitaxial in nature. Figure 2(b) shows the clear and distinct (200) set of peaks of BZT, BCT, SRO and STO at 44.6°, 45.1°, 45.75° and 46.47°, respectively.

The diffraction peak positions (2θ) of (200) reflections were obtained by Gaussian fitting. We did not notice any extra reflection peaks, which confirm that these films are phase pure. This means Ca^2+^ and Zr^4+^ are completely diffused into the BaTiO_3_ crystal lattice to form a homogenous solid solution.

### Morphological characterization

**Figure 3:**
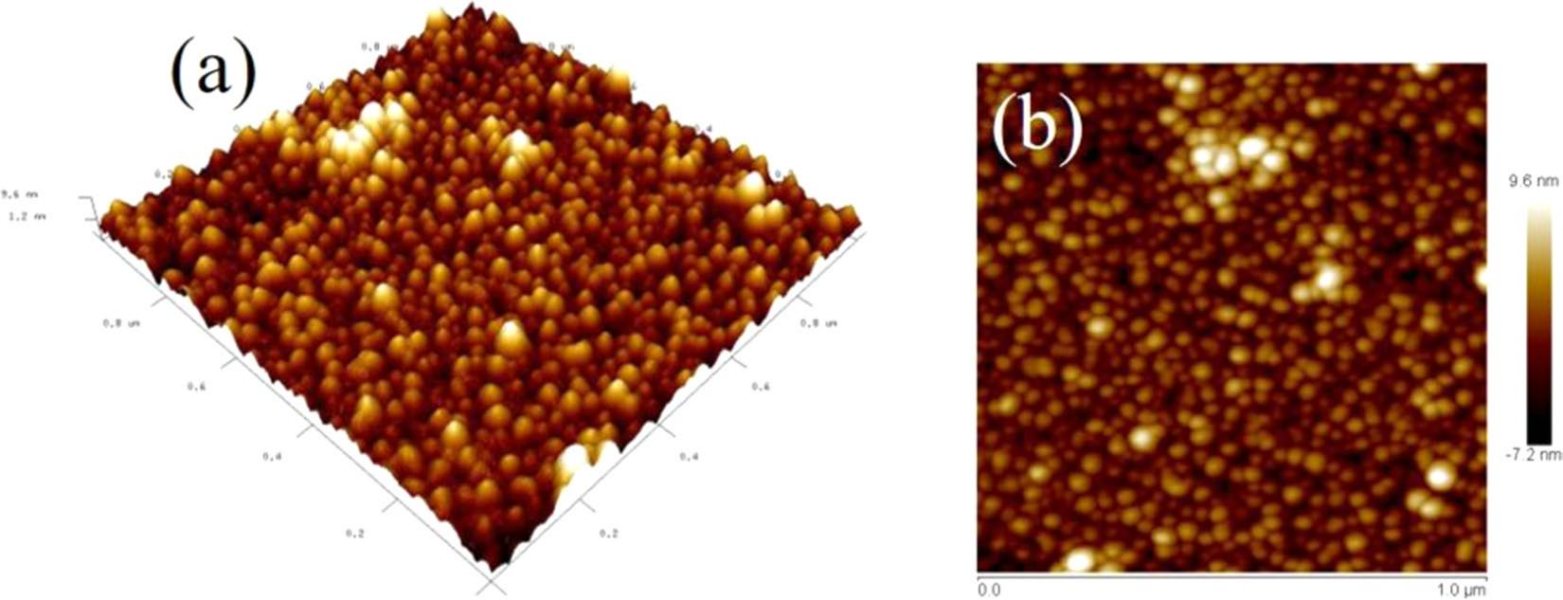
AFM images of BZT/BCT heterostructures: 3D (**a**) and 2D (**b**).

The surface morphology of the top layer of BZT/BCT heterostructure grown on SRO buffered STO substrate is shown in Fig. 3(a,b). The atomic force microscopy (AFM) images were captured in the tapping mode on a scan size of 1 × 1 *μ*m^2^. It can be seen that these nanostructures exhibit highly dense, well-connected granular structure, with average surface roughness (*R*_a_) 2 nm. The three-dimensional (3D) AFM image shown in Fig. 3(a) indicates that surface of these heterostructures are smooth and homogeneous, indicating high quality thin films due to epitaxial growth. Such a highly dense smooth surface with low roughness is favorable for improvement of ferroelectric, dielectric and electrical properties. Figure 3(b) shows the two-dimensional (2D) AFM images on a scan size of 1 × 1 *μ*m^2^ area, which mentions smooth morphologies with uniform grain size ∼40 nm. The thickness of these thin films was estimated to be ~260 nm using profilometer line scan.

### Electrical, dielectric and ferroelectric properties of BZT and BCT heterostructures

The reduced leakage current of the ferroelectric materials is very important to get the enhanced dielectric, ferroelectric and other functional properties with higher breakdown field^25,43^. The effective way to reduce leakage current is to suppress oxygen vacancies (main charge carriers of ferroelectric materials) during film deposition. In our work, a lower oxygen atmosphere of 20 mTorr is used to deposit SRO film as a bottom electrode and a higher oxygen atmosphere of 100 mTorr is used for the growth of BCT and BZT thin films as a dielectric layer. To reduce the oxygen vacancies all the heterostructures were annealed at 300 Torr of oxygen atmosphere after deposition. More oxygen atmosphere means less oxygen vacancies, and hence high resistivity of the film as each oxygen vacancy provides two electrons.

**Figure 4:**
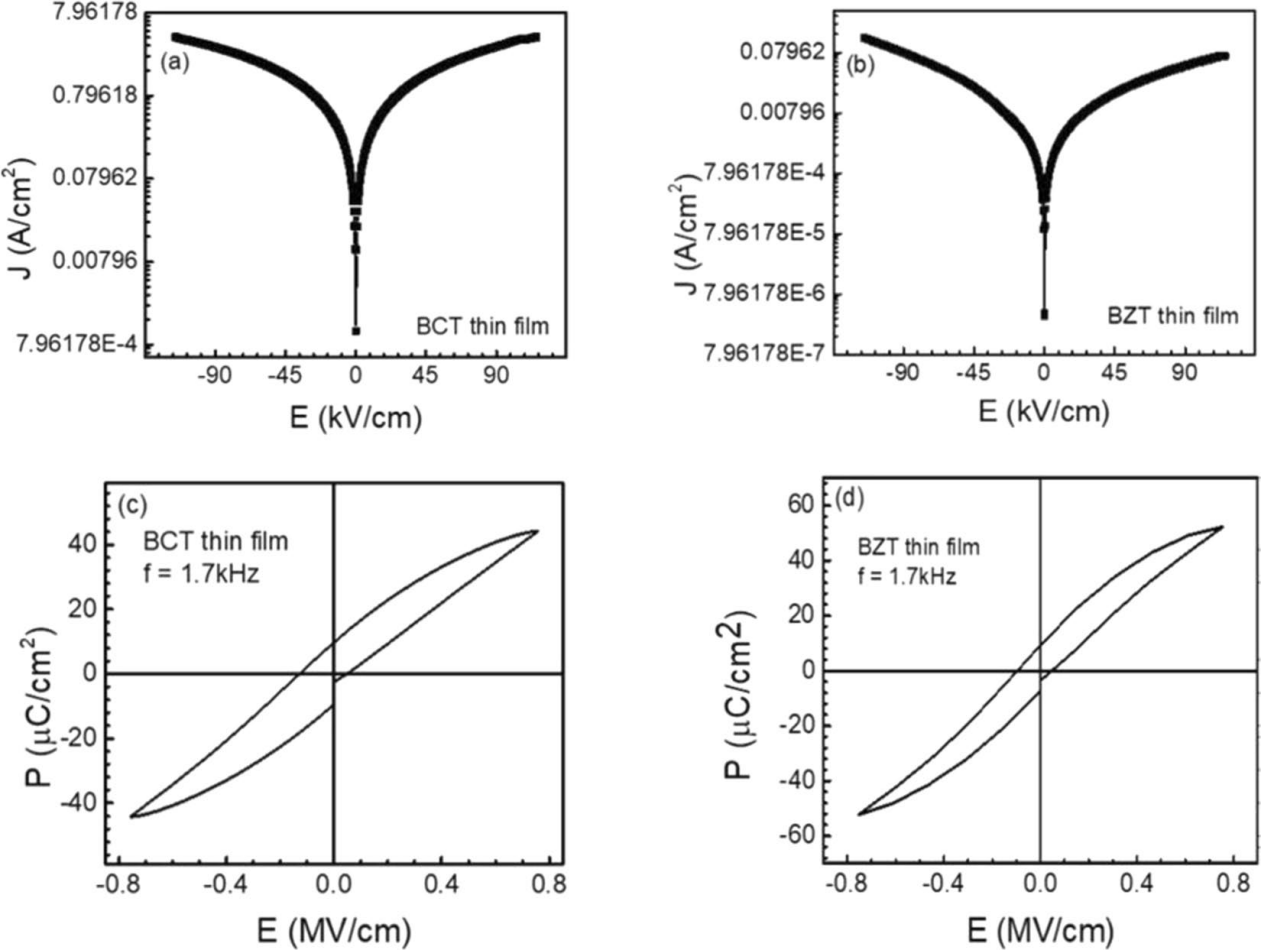
Current density-electric field (J-E) curves for BCT thin film (**a**) and for BZT thin film (**b**), and electric field dependent polarization (**c**) and (**d**) for BCT and BZT thin film, respectively.

Figure 4(a,b) show the room temperature current density versus electric field (J-E) curves of up to 117 kV/cm, where both the J-E curves show low leakage current and symmetrical behavior under positive and negative applied electric fields. It is worth noting that there is an exponential increase of current with voltage at low voltages, followed by almost saturation at high voltages. For the BCT thin film, the leakage current increases from 10^−4^ A/cm^2^ to 7.9 A/cm^2^, whereas for the BZT thin film, it increases from 10^−6^ A/cm^2^ to 0.08 A/cm^2^ at 0 and 117 kV/cm applied field, respectively. It can be seen from the Eq. (1) that polarization is a necessary parameter in determining the energy storage performance. Figure 4(c,d) display the P-E curves of BCT and BZT thin films, respectively at 1.7 kHz at room temperature. Well-defined saturated hysteresis curves were observed for both BCT and BZT thin films with low remnant polarization (P_r_) of 10 µC/cm^2^ and low coercive field of 0.05 MV/cm, which confirms the films are ferroelectric in nature.

### Electrical, dielectric and ferroelectric properties of BZT/BCT heterostructures

**Figure 5:**
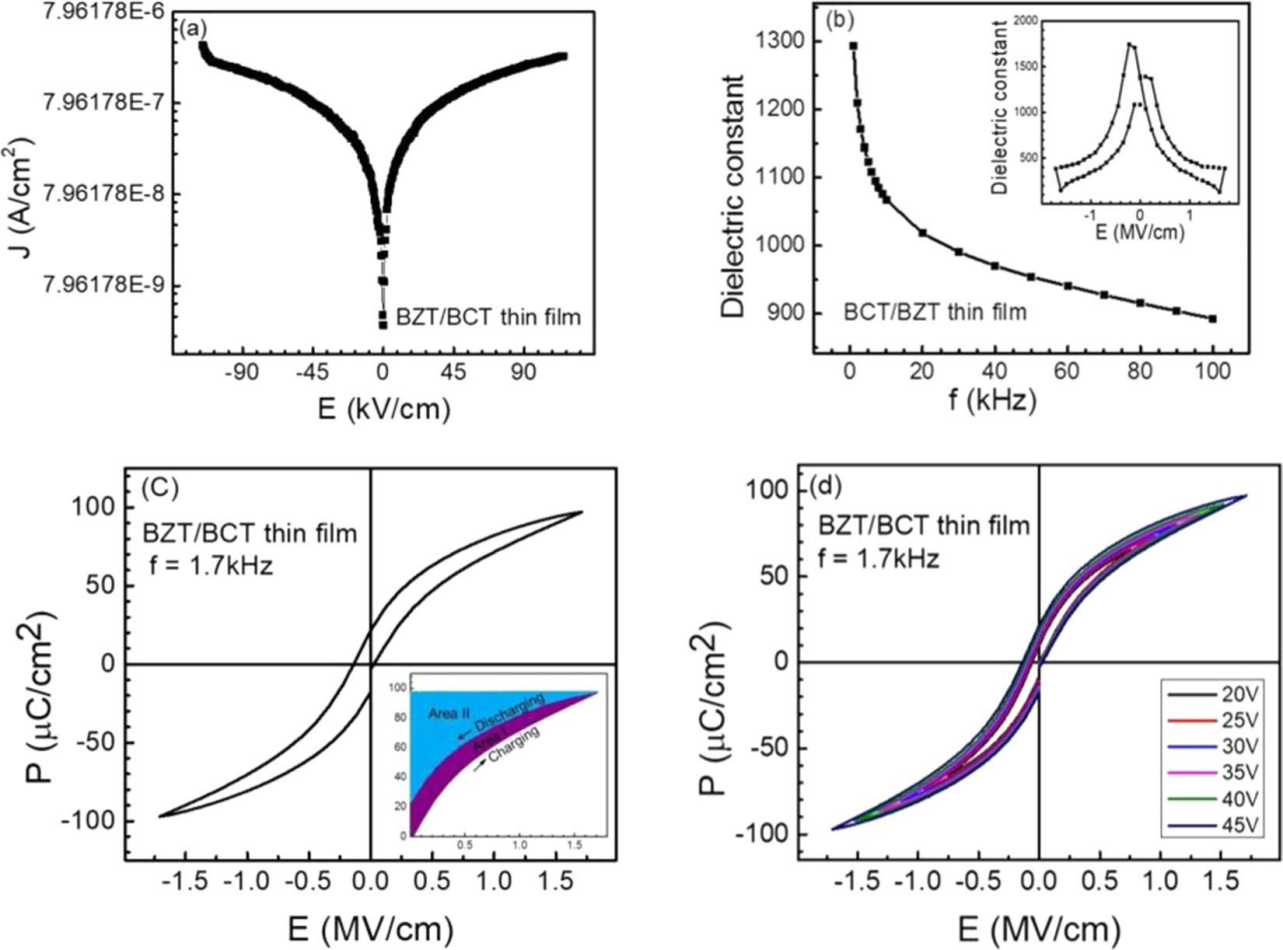
Variation of (**a**) leakage current density with electric field, (**b**) dielectric constant as a function of frequency and electric field (in inset), (**c**) electric field dependent polarization and (**d**) electric field dependent polarization at various applied voltages for BCT/BZT thin film. The inset of (**c**) shows the charge-discharge cycle where area I (purple shaded area) corresponds to the energy loss density and area II (blue shaded area) is the discharged energy density.

Figure 5(a) shows the room temperature J-E curve of BCT/BZT heterostructures where the leakage current is greatly reduced (<10^−9^ A/cm^2^) compared to individual BZT and BCT thin films. It is well known that a low leakage current helps to achieve high polarization and high breakdown field. One can notice that all the J-E curves follow Ohm’s law at low electric fields and almost saturate at high electric fields. As the electric field increases, the conduction mechanism changes due to different sources of charge carriers. For example, space-charge limited conduction becomes dominant at medium fields whereas the Schottky-Frankel conduction dominates at high fields^44,45^.

It is obvious that the dielectric constant is one of the major factors that affects energy storage performance. The dielectric constant of BCT/BZT heterostructure is observed to decrease with the increase of frequencies, as depicted in Fig. 5b, indicating a typical characteristic of ferroelectric materials. The main contributions in the dielectric constant of solids, are due to existence of four types of polarizations: (i) dipolar polarization, (ii) space-charge/interfacial polarization, (iii) electronic polarization and (iv) ionic polarization^46^. The higher dielectric constant at lower frequencies are due to the existence of all types of polarizations. It is clearly seen that the dielectric constant of the BCT/BZT thin film, at a low frequency of 1 kHz, is found to be ~1,300, which is higher than the dielectric constant of individual BZT (~557) and BCT (~325) films (not shown here). This higher dielectric constant is attributed to the contribution of interfacial polarization observed at the multiple interfaces and perfect electromechanical coupling between the BCT and BZT layers due to high epitaxy^35^. The higher the dielectric constant, the higher the breakdown field strength will be. Therefore, improvement on the electric insulation of the thin film is one of the important factors for the large energy performance in dielectric capacitor as it allows a larger electric field and higher polarization.

The effect of dc electric field on the dielectric constant is usually measured from capacitance-voltage (C-V) characteristics to observe the ferroelectric nature of BZT/BCT thin film. A typical characteristic C-V butterfly curve measured at different dc bias fields (±117 kV/cm) at room temperature suggests a considerable dielectric tunability with dc bias field, as shown in inset of Fig. 5(b), and this variation is associated with domain process^47^. At low dc electric field, the dielectric constant is maximum, which is due to a large change in polarization because of domain reversal^8^. However, at a high dc field, most of the dipoles are aligned along the direction of the dc field and hence a relatively small value of dielectric constant is observed due to the vibration of the dipoles^8^. A little asymmetry in C-V curve is also observed due to the different types of top and bottom electrodes in our metal-ferroelectric-metal structure (Au/(BZT/BCT)/SRO).

Figure 5(c) exhibits slim and well-saturated P-E loops of BCT/BZT heterostructure at 1.7 kHz at room temperature, which confirms its ferroelectric nature. We noticed large polarization, and this is larger than the single layer of BZT and BCT having remnant and saturation polarization of 20 and 100 µC/cm^2^, respectively, at applied field of 1.731 MV/cm. In these BZT/BCT heterostructures, interfacial polarization plays very important role in addition to other polarizations. At the BZT/BCT interface, significant amount of space charges were generated and these interfacial charges behave as effective charge traps for moving electrons from metal electrodes under the high applied electric field^42^, which increases the electrical resistivity and enhances the breakdown field strength, polarization and electrical insulation. Moreover, the observed strain at the multiple interface and the perfect strain transmission between the layers, slow down the spread and growth of the significant electric path under high applied electric field^35^. Our results indicate that a heterostructure having multiple interfaces increases its electrical insulation and hence enhances the polarization.

The charging and discharging energy densities of the dielectric capacitors are calculated from the integral of the area enclosed by charge/discharge curve and y-axis^8,48^ using P-E loops via Eq. (1). Areas I (purple-shaded) and II (blue-shaded) represent the charging energy density, whereas area II represents discharging energy density (inset of Fig. 5(c)). The P-E loops of the heterostructure, at different applied voltages from 20 to 45 V, are utilized to determine the electrical storage properties, Fig. 5(d). It is clearly seen that, even at higher applied voltage, the films exhibit slim and well-saturated P-E loops, which signify the optimistic energy storage performances. Lastly, the electrical energy storage efficiency is calculated using Eq. (2). The energy density and efficiency of BCT/BZT dielectric capacitor, at various applied electric fields, are summarized in Table 1.

**Table 1:** Energy density and efficiency of BCT/BZT thin film at various applied electric fields.

Electric field (MV/cm)	Charge energy density (J/cm^3^)	Discharge energy density (J/cm^3^)	Efficiency (η) %
0.769	29.87	15.54	52.2
0.962	42.74	20.85	48.8
1.154	56.72	26.39	46.5
1.346	71.78	32.11	44.7
1.538	87.54	37.85	43.2
1.731	97.13	42.10	43.3

**Figure 6:**
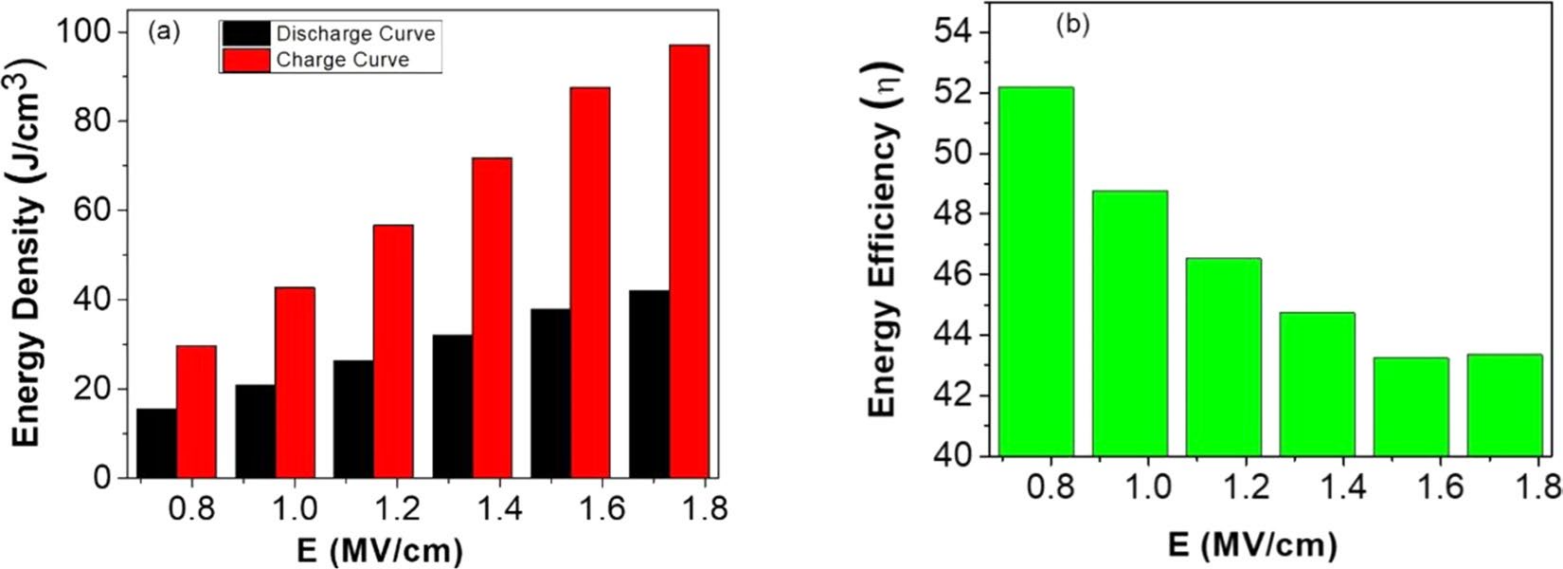
Electric field dependent charge and discharge energy densities (**a**) and efficiencies (**b**) of BCT/BZT thin film.

Figure 6(a) presents external electric field-dependent charge and discharge energy densities of BZT/BCT thin films where the charging and discharging energy densities increase linearly with the electric field. The maximum values of discharge and charge energy densities are found to be 42.10 J/cm^3^ and 97.13 J/cm^3^, respectively, at maximum applied field of 1.731 MVcm^−1^. These higher values of discharge and charge energy densities might be due to the large difference between maximum polarization (P_max_) and remnant polarization (P_r_) and high breakdown field strength. Similarly, the efficiency (η) of the heterostructure was calculated using Eq. (2) and found to vary from 43.3% up to 52.2% with varying electric fields. Even though the charging and discharging energy densities of the heterostructure increase linearly with the increase in applied electric fields, its efficiency gradually declined from 52.2% to 43.3% because of relatively larger P_r_ and higher leakage current at high electric fields, which is displayed in Fig. 6(b).

The electrical energy storage performance of various lead-free materials was studied. Recently, a giant recoverable energy-storage density of 39.11 J/cm^3^ was reported in BCT-BZT composite relaxor-ferroelectric at 2.08 MV/cm by Puli *et al*.^8^ Similarly, the discharge energy density of 12.24 J/cm^3^ at 1.65 MV/cm has reported by Ortega *et al*.^3^ on relaxor-ferroelectric multilayer BT/BST and Pan *et al*.^49^ has reported maximum energy density of 51 J/cm^3^ in Mn-doped 0.4BiFeO_3_–0.6SrTiO_3_ (BFSTO) thin film capacitor at 3.6 MV/cm. However, our lead-free relaxor-ferroelectric BCT/BZT multilayers demonstrate excellent discharging and charging energy densities with high efficiency at very low voltage. The present results evidently show that the energy densities of BCT/BZT multilayers are highly promising contenders to those of other reported lead-free systems, at low voltages, and rival the lead-based materials.

## Conclusions

The BZT/BCT heterostructures were grown successfully on SRO buffered STO single crystal substrate by optimized PLD. The diffraction peaks from the substrate only and their pseudo cubic reflections (*l00*) confirming the phase purity, the high crystallinity, and the epitaxial nature of the heterostructures. The surface morphology captured by AFM in tapping mode indicates the high-quality film growth with low surface roughness. Our BZT/BCT heterostructures exhibit promising ferroelectric and dielectric properties with high charge and discharge energy densities at low voltage compared to similar materials. The above results demonstrate that the favorable relaxor-ferroelectric property, large polarization, together with enhanced breakdown strengths, give rise to large energy storage density of ∼42.1 J/cm^3^ in the BCT/BZT heterostructure at applied electric field 1.73 MV/cm, which are superior to other reported lead-free systems, at low voltage, and rival the best lead-based systems. Therefore, the careful interface engineering, perfect strain transmission by epitaxial growth of ferroelectric systems are an effective way in tuning/improving discharging and charging energy density characteristics, which may be useful for both high power and energy density applications.
